# Radiation dose escalation for loco-regional recurrence of breast cancer after mastectomy

**DOI:** 10.1186/1748-717X-8-13

**Published:** 2013-01-11

**Authors:** Heath D Skinner, Eric A Strom, Sabin B Motwani, Wendy A Woodward, Marjorie C Green, Gildy Babiera, Daniel J Booser, Funda Meric-Bernstam, Thomas A Buchholz

**Affiliations:** 1 Department of Radiation Oncology, The University of Texas M.D. Anderson Cancer Center, Houston, TX 77030, USA; 2 Department of Breast Medical Oncology, The University of Texas M.D. Anderson Cancer Center, Houston, TX 77030, USA; 3 Department of Surgical Oncology, The University of Texas M.D. Anderson Cancer Center, Houston, TX 77030, USA; 4 Department of Radiation Oncology, Robert Wood Johnson Medical School, Cancer Institute of New Jersey, New Brunswick, NJ, 08901, USA

**Keywords:** Breast cancer, Radiation, Local recurrence, Dose escalation

## Abstract

**Background:**

Radiation is a standard component of treatment for patients with locoregional recurrence (LRR) of breast cancer following mastectomy. The current study reports the results of a 10% radiation dose escalation in these patients.

**Methods:**

159 patients treated at MD Anderson Cancer Center between 1994-2006 with isolated LRR after mastectomy alone were reviewed. Patients in the standard treatment group (65 pts, 40.9%) were treated to 50 Gy comprehensively plus a boost of 10 Gy. The dose escalated group (94 pts, 59.1%) was treated to 54 Gy comprehensively and a minimum 12 Gy boost. Median dose in the standard dose and dose escalated group was 60 Gy (±1 Gy, 95% CI) and 66 Gy (±0.5 Gy, 95% CI) respectively. Median follow up for living patients was 94 months from time of recurrence.

**Results:**

The actuarial five year locoregional control (LRC) rate was 77% for the entire study population. The five year overall survival and disease-free survival was 55% and 41%, respectively. On multivariate analysis, initial tumor size (p = 0.03), time to initial LRR (p = 0.03), absence of gross tumor at the time of radiation (p = 0.001) and Her2 status (p = 0.03) were associated with improved LRC. Five year LRC rates were similar in patients with a complete response to chemotherapy without surgery and patients with a complete surgical excision (77% vs 83%, p = NS), compared to a 63% LRC rate in patients with gross disease at the time of radiation (p = 0.024). LRC rates were 80% in the standard dose group and 75% in the dose escalated group (p = NS).

**Conclusions:**

While LRR following mastectomy is potentially curable, distant metastasis and local control rates remain suboptimal. Radiation dose escalation did not appear to improve LRC. Given significant local failure rates, these patients are good candidates for additional strategies to improve their outcomes.

## Background

The therapeutic approaches for isolated locoregional recurrence (LRR) of breast cancer vary widely from palliation to aggressive multimodality salvage therapy. In those patients with isolated LRR after mastectomy treated with curative intent, long-term survival ranges from as low as 25% [1] to a more typical 50% [2-6]. The choice of therapies is complicated by the lack of a place in the current staging system for these patients, with many patients assigned a “Stage IV” designation despite having disease that is potentially curable and without evidence of distant metastasis. Previously, we and others have shown that patients with isolated LRR can be cured, but there remains considerable debate regarding the best combination of local and systemic treatments [2,3,7,8]. For definitive treatment, optimum management appears to involve systemic therapy, surgical resection of the recurrence when feasible, and post-operative radiation. However, even with aggressive local therapy, achieving durable local control is challenging [2,9,10].

In early breast cancer, dose escalation via the addition of a tumor bed boost has been shown to result in a local control benefit [11]. Similarly, we have seen improved local control in inflammatory breast cancer with the use of radiation dose escalation [12]. These findings led us to implement a systematic 10% radiation dose escalation in patients treated for isolated locoregionally recurrent breast cancer to improve on our previously reported results. The vast majority of patients following this practice change were treated to the higher dose, with exceptions due to patient preference or other factors. This study compares patient outcomes using our previous standard dose (typically 60 Gy) versus our current escalated dose (66 Gy).

## Methods

After obtaining MD Anderson Cancer Center Institutional Review Board (IRB) approval for a retrospective chart analysis, a total of 292 charts was reviewed, representing all patients identified has having received radiotherapy for loco-regionally recurrent breast cancer after mastectomy between 1994 - 2006 at MDACC. 133 patients were excluded from this study because they had visceral or bony metastases at time of recurrence, previous radiation to the breast or chest wall, or pathology other than breast cancer, leaving a total of 159 patients who were treated with curative intent. The data from these patients were analyzed for this study.

These patients were then divided into two groups: 65 patients who were treated the lower dose and 94 patients treated using the escalated dose schema. Patient characteristics at the time of initial diagnosis of breast cancer, stratified by radiation treatment group are presented in Table 1. The initial surgical treatment was a modified radical mastectomy in 141 patients (89%), while 18 patients (11%) were treated with total mastectomy and sentinel lymph node biopsy. The majority of patients (72%) had received some form of systemic therapy for their initial diagnosis of breast cancer consisting of either hormonal therapy alone (10%), chemotherapy alone (42%) or a combination of the two (20%). Most patients who had not received systemic therapy at initial diagnosis had presented with low volume (T1-T2), ER negative and node negative disease.

**Table 1:** Patient characteristics at initial diagnosis of breast cancer

				**Dose (Gy)**				
		**All patients**		**<66**		**≥66**		
		**Median**	**Range**	**Median**	**Range**	**Median**	**Range**	
**Age at initial diagnosis (years)**		46	25–77	47	26–77	44	25–77	
**Age at LRR (years)**		48	25–78	50	26–78	46.5	25–77	
**F/U from initial diagnosis (mos)**		145	6–390	140	20–382	148	6–390	
**F/U from XRT**		94	1–218	108	1–218	92	4–164	
**Stage**		**n**	**%**	**n**	**%**	**n**	**%**	**p-value**
	T1	55	34.6%	26	40.0%	29	30.9%	**NS**
	T2	76	47.8%	31	47.7%	45	47.9%	
	T3	14	8.8%	3	4.6%	11	11.7%	
	T4	7	4.4%	2	3.1%	5	5.3%	
	TX	7	4.4%	3	4.6%	4	4.3%	
	N0	81	50.9%	34	52.3%	47	50.0%	**NS**
	N1	52	32.7%	23	35.4%	29	30.9%	
	N2	14	8.8%	6	9.2%	8	8.5%	
	N3	8	5.0%	1	1.5%	7	7.4%	
	NX	4	2.5%	1	1.5%	3	3.2%	
**Primary histopathology**								
	IDC	141	88.7%	56	86.2%	85	90.4%	**NS**
	ILC	18	11.3%	9	13.8%	9	9.6%	
**Tumor Grade**								
	Well Diff	10	6.3%	2	3.1%	8	8.5%	**NS**
	Mod Diff	43	27.0%	17	26.2%	26	27.7%	
	Poor Diff	86	54.1%	37	56.9%	49	52.1%	
	Unk	20	12.6%	9	13.8%	11	11.7%	
**Markers**								
	**ER**							
	Negative	70	44.0%	34	52.3%	36	38.3%	**0.008**
	Positive	69	43.4%	19	29.2%	50	53.2%	
	Not done/Unknown	20	12.6%	12	18.5%	8	8.5%	
	**PR**							
	Negative	69	43.4%	32	49.2%	37	39.4%	**NS**
	Positive	65	40.9%	20	30.8%	45	47.9%	
	Not done/Unknown	25	15.7%	13	20.0%	12	12.8%	
	**Her2**							
	Negative	30	18.9%	10	15.4%	20	21.3%	**NS**
	Positive	15	9.4%	5	7.7%	10	10.6%	
	Unknown/not done	114	71.7%	50	76.9%	64	68.1%	
**Initial systemic therapy**								
	Hormonal therapy only	16	10.1%	7	10.8%	9	9.6%	**0.05**
	Chemotherapy only	66	41.5%	21	32.3%	45	47.9%	
	Chemotherapy and hormonal therapy	32	20.1%	11	16.9%	21	22.3%	
	None	45	28.3%	26	40.0%	19	20.2%	
**Initial surgery**								
	MRM	141	88.7%	59	90.8%	82	87.2%	**NS**
	Other	18	11.3%	6	9.2%	12	12.8%	
**Menopausal status**								
	Pre	67	42.1%	28	43.1%	39	41.5%	**NS**
	Peri	18	11.3%	5	7.7%	13	13.8%	
	Post	63	39.6%	27	41.5%	36	38.3%	
	Unknown	11	6.9%	5	7.7%	6	6.4%	
**Race**								
	Asian	11	6.9%	6	9.2%	5	5.3%	**NS**
	Black	27	17.0%	7	10.8%	20	21.3%	
	Hispanic	19	11.9%	11	16.9%	8	8.5%	
	Other	2	1.3%	1	1.5%	1	1.1%	
	White	100	62.9%	40	61.5%	60	63.8%	

Comparison between escalated (≥66) and standard dose (<66) groups at time of initial treatment. ER positivity was significantly less in the standard dose group (p = 0.008). No other significant differences in patient characteristics at diagnosis between groups were seen.

All patients with recurrences in the chest wall or reconstructed breast were treated with surgical resection if possible. When surgical resection was not feasible, patients were treated with systemic chemotherapy initially to downstage the tumor and allow for surgical resection. Patients who had a complete clinical response to initial chemotherapy were treated with radiotherapy alone. The majority of patients (~70%) received chemotherapy either prior to or after resection for their LRR. Following resection or chemotherapy, patients were treated with radiation. Patients with ER + disease and those with HER2+ disease frequently received hormonal therapy and/or trastuzumab (if treated in the era when such treatments were approved).

The radiotherapy fields used for the treatment of recurrent disease were designed to encompass the entire chest wall and the regional lymphatics. Several techniques were used to accomplish this goal. Treatment to the chest wall was delivered using either photon tangential fields or appositional electrons. The supraclavicular fossa and axillary apex were treated with an anterior photon field. A separate appositional electron field was commonly used to treat the medial chest wall and internal mammary nodes. Although omitted by some institutions, in our practice a boost was consistently delivered to the chest wall flaps including the scar with generous margin and any additional sites of disease.

In the standard dose cohort, the primary radiation fields were treated to 50 Gy followed by a 10 Gy boost to the chest wall flaps and any additional sites of recurrence. The dose escalated cohort had the primary radiation fields treated to 54 Gy followed by a 12 Gy boost (Table 2).

**Table 2:** Characteristics of loco-regional recurrence of breast cancer and subsequent treatment

			**Dose (Gy)**				
	**All patients**		**<66**		**≥66**		
**Median time to recurrence (mos)**	26		23.5		32.0		**NS**
**Range**	2–303		5–241		2–303		
**Recurrence site**	**n**	**%**	**N**	**%**	**n**	**%**	
Axilla alone	12	7.5%	7	10.8%	5	5.3%	**NS**
Reconstructed breast/CW alone	100	62.9%	39	60.0%	61	64.9%	
SCV alone	12	7.5%	6	9.2%	6	6.4%	
ICV alone	2	1.3%	1	1.5%	1	1.1%	
IMC alone	1	0.6%	1	1.5%	0	0.0%	
Sternum alone	1	0.6%	0	0.0%	1	1.1%	
Reconstructed breast/CW and lymph nodes	23	14.5%	8	12.3%	15	16.0%	
Multiple nodal sites	8	5.0%	3	4.6%	5	5.3%	
**Multiple sites of recurrence**							
Yes	31	19.5%	11	16.9%	20	21.3%	**NS**
No	128	80.5%	54	83.1%	74	78.7%	
**Systemic therapy for recurrence**							
**Hormones alone**	34	21.4%	13	20.0%	21	22.3%	**NS**
**Chemotherapy +/- hormones**	111	69.8%	45	69.2%	66	70.2%	
**None**	12	7.5%	6	9.2%	6	6.4%	
**Unknown**	2	1.3%	1	1.5%	1	1.1%	
**Surgery for recurrence**							
Yes	118	74.2%	51	78.5%	67	71.3%	**NS**
No	41	25.8%	14	21.5%	27	28.7%	
**Gross tumor at time of radiation**							
Yes	42	26.4%	20	30.3%	22	26.4%	**NS**
No	117	73.6%	45	69.2%	72	73.6%	
		**SD**		**SD**		**SD**	
**Median dose (Gy)**	63.5	4.4	60.0	3.6	66.0	2.0	
**Median microscopic dose (Gy)**	51.2	2.6	50.0	2.3	52.3	2.3	
**Median boost dose (Gy)**	12.9	3.2	10.0	2.2	14.0	3.1	

Recurrent disease and its treatment was similar in both escalated (≥66) and standard (<66) dose groups.

As radiotherapy was the final aspect of multimodality therapy for nearly all patients, all outcomes are measured from the completion date of radiotherapy. The primary outcome was loco-regional control (LRC), which was defined as freedom from clinical or radiographic evidence of recurrence of breast cancer within the ipsilateral chest wall, sternum or draining lymphatics after treatment with radiation. Secondary outcomes included overall survival (OS), disease free survival (DFS) and distant metastasis free survival (DMFS). DFS after radiation was defined as the interval of time without LRR, distant metastasis (DM) or death following completion of radiation. DMFS after radiation was defined as the interval of time without distant metastasis (DM) or death following completion of radiation. OS after radiation was defined as the time from the completion of radiation to death. A starting point at the completion of radiation was used to create uniformity between radiation dose groups. Median follow-up from the time of recurrence in living patients was 108 and 92 months for standard and dose-escalated groups respectively. Statistics were performed using SPSS software (v16.0). Dose group differences were determined using the Chi-squared statistic. The probabilities of LRC, DFS, DMFS and OS were calculated using the Kaplan-Meier method and log rank statistics. Univariate and multivariate analysis was performed using forward step-wise Cox regression analysis. All p values were two-sided and if less than 0.05 were deemed significant.

## Results

### Outcomes for the study population

The 5 year actuarial locoregional control rate following radiation for the entire study population was 77%, with 31/159 patients failing to secure locoregional control after multimodality therapy. The most common sites of subsequent recurrence following radiation included: chest wall or reconstructed chest wall (21), sternum (2), axilla (6), supraclavicular basin (SCV) (6), internal mammary chain (IMC) (1), and infraclavicular basin (ICV) (1) [Table 3]. Six patients had multiple sites of recurrence after treatment. All locoregional failures after radiation occurred either in field (27 patients) or at the field margin (4 patients). Treatment failures were occasionally seen on the anterior abdomen, contralateral chest wall or lymph nodes or posterior thorax, but were classified as distant spread. On univariate analysis, multiple factors were associated with recurrence following radiation (Additional file 1: Table S1). However, on multivariate analysis, only initial tumor size (p = 0.03), time to initial recurrence (p = 0.03), clinically detectable disease at the time of radiation (p = 0.001) and Her2 status (p = 0.03) were associated with locoregional failure following radiotherapy (Table 4).

**Table 3:** Subsequent recurrence after treatment for loco-regional recurrence in breast cancer

			**Dose (Gy)**			
	**All patents**		**<66**		**≥66**	
	**n**	**%**	**n**	**%**	**n**	**%**
**Number of patients**	28	100.0%	12	42.9%	16	57.1%
**Site**						
In Field	25	89.3%	10	35.7%	15	53.6%
Field margin	3	10.7%	2	7.1%	1	3.6%
Chest Wall	19	67.9%	8	28.6%	11	39.3%
Sternum	2	7.1%	1	3.6%	1	3.6%
Axilla	6	21.4%	4	14.3%	2	7.1%
Supraclav fossa	6	21.4%	2	7.1%	4	14.3%
Reconstructed breast	3	10.7%	1	3.6%	2	7.1%
Internal Mammary	1	3.6%	1	3.6%	0	0.0%
Infraclav fossa	1	3.6%	0	0.0%	1	3.6%
**Progression during XRT**	2	7.1%	1	3.6%	1	3.6%

No difference was found between escalated (≥66) and standard (<66) dose groups in number or site of recurrences. Six patients had synchronous loco-regional recurrences. All recurrences were either in field or at the field margin. The sum of the anatomic sites exceeds 100% to account for simultaneous recurrences.

**Table 4:** Multivariate analysis of outcomes

**LRR after XRT**	**Comparison**	**RR**	**p-value**
**·Primary diagnosis**			
Size	Continous	1.29	0.02
**·Recurrence**			
Time to LRR	Continous	0.97	0.03
Gross tumor at XRT	Yes vs. No	18.15	0.001
Her 2 status	Positive vs. Negative	5.00	0.03
**OS after XRT**	**Comparison**	**RR**	**p-value**
**·Primary diagnosis**			
Tumor stage	T2-4 vs. T1	2.54	0.001
Positive Nodes	Positive vs. Negative	2.49	0.01
Nodal LRR	Yes vs. No	1.96	0.05
**·Recurrence**			
ER status	Positive vs. Negative	0.37	0.005
Gross tumor at XRT	Yes vs. No	1.95	0.002
**DFS after XRT**	**Comparison**	**RR**	**p-value**
**·Primary diagnosis**			
Percent Positive Nodes	<25% vs. Negative	1.79	0.03
	≥25% vs. <25%	2.73	<0.001
Tumor stage	T2-4 vs. T1	2.02	0.01
**·Recurrence**			
Gross tumor at XRT	Yes vs. No	1.79	0.02
**DMFS after XRT**	**Comparison**	**RR**	**p-value**
**·Primary diagnosis**			
Percent Positive Nodes	<25% vs. Negative	2.30	0.003
	≥25% vs. <25%	3.40	<0.001
**·Recurrence**			
Size	≥2 cm vs. < 2 cm	1.63	0.05
Gross tumor at XRT	Yes vs. No	2.07	0.003

Five year OS, DFS and DMFS after radiation was 55%, 41% and 44% respectively for the entire study population, with 83 patients eventually developing distant metastatic disease. Multiple patient and treatment factors significantly affected OS, DFS and DMFS after radiation in this study on univariate analysis (Additional file 1: Table S1). For example, those patients who had a surgical resection of their recurrence had significantly improved OS (p = 0.05) and DFS (p = 0.01) after radiation. However, on multivariate analysis only the presence of gross disease at the time of radiation was uniformly predictive of poorer OS (p = 0.002), DFS (p = 0.02) and DMFS (p = 0.003). Importantly, in the 31 patients who failed locally or regionally following radiation, five year OS was 27% compared to 62% in the remaining patients (p = 0.002). Also, 5 year DMFS was significantly reduced in patients with failure following radiation compared to those patients who achieved local control (16% vs. 51%, p = 0.002).

### The presence of gross disease as a prognostic factor

In patients with clinically apparent disease at the time of radiation, five year LRC was 63% compared to 81% in patients with no residual gross disease (p = 0.019). Interestingly, those patients who had a clinical complete response (CR) to chemotherapy, but no surgical resection had comparable loco-regional control rates to those patients whose tumor was removed by surgical excision (77% vs. 83% p = NS). Furthermore, five year OS, DFS and DMFS in the patients with gross disease at the time of radiation was 34%, 19% and 21% compared to 62%, 50% and 52% in patients with no gross disease (p = 0.000007, p = 0.000007, p = 0.00002).

### The impact of dose escalation on outcome

The standard and dose escalated treatment groups had no significant differences in initial tumor or nodal stage, age at initial diagnosis, or menopausal status (Table 1). A significantly greater number of patients in the standard dose group had ER-negative initial tumors (52.3% vs. 38.3%, p = 0.007). Patients in the dose escalated group were somewhat more likely to receive systemic therapy for their initial diagnosis (60% vs. 79.8%, p = 0.05). Patient and tumor characteristics at the time of loco-regional recurrence (LRR) are presented in Table 2 and were generally comparable. Median time to recurrence was not significantly different between the groups (24 vs. 34 mos). The majority of isolated LRR were seen in the chest wall or reconstructed chest wall either alone (62.9%) or a combination of chest wall and nodal recurrence (14.5%). No significant differences were seen between groups in regards to site of recurrence. Similar numbers of patients has gross residual disease at the time of radiation (30.8% vs. 23.4%, p = NS), defined as clinical or radiographic evidence of residual disease. The use of systemic chemotherapy (69.2% vs. 70.2%, p = NS), and surgical resection (78.5% vs. 71.3%, p = NS) was not significantly different between the two dose groups. However, slightly more patients in the dose escalated group received hormonal therapy (38.5% vs. 57.4%, p = 0.05), reflecting a larger percentage of the population being ER-positive (24.6 vs. 43.6, p = 0.05). Most patients (91.2%) were treated with some form of systemic therapy, either cytotoxic chemotherapy, hormonal therapy or a combination of the two.

While radiation dose escalation was intended to improve loco-regional control, LRC was 80% in the standard dose group compared to 75% in the dose escalated group (p = 0.94). Furthermore, LRC in patients with gross disease at the time of XRT was comparable between dose groups (56% vs. 71%, p = 0.27). LRC in patients without gross disease was also similar between dose groups (83% vs. 80%, p = NS).

**Figure 1:**
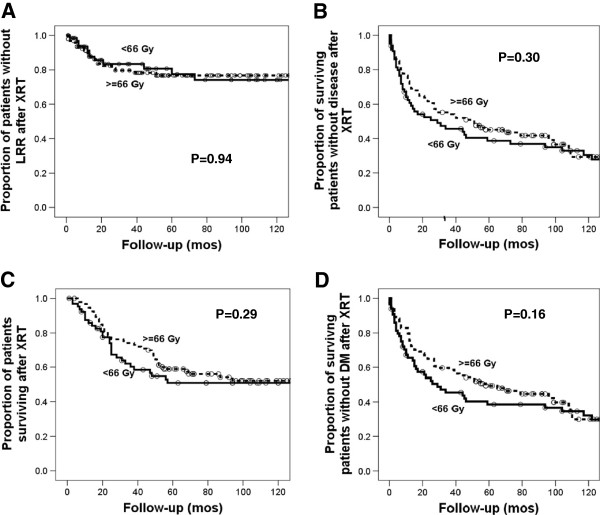
**Survival and loco-regional recurrence after treatment for loco-regionally recurrent breast cancer. **No significant difference was seen between standard and dose-escalated groups in LRR (**A**), DFS (**B**), OS (**C**) or DMFS (**D**) after XRT.

Five year OS after radiation was 52% in the standard dose group compared to 57% in the dose escalated group (p = 0.29), while five year DFS was 39% in the standard dose group compared to 43% in the dose escalated group (p = 0.3) (Figure 1). A trend toward improved DMFS was seen in the dose escalated patients, however this was not significant (39% vs. 47%, p = 0.16). Nor did dose escalation improve any survival outcome in patients with gross disease at the time of radiation.

### Complications and toxicity

Complications and toxicity directly attributable to radiation were difficult to assess due to the multidisciplinary nature of the treatment for locoregional recurrence and the presence of pre-existing treatment effects. Complications clearly due to therapy received for the initial cancer diagnosis were excluded, unless this significantly changed after completion of radiotherapy. A total of 8 patients in this study developed a grade 3 or greater complications attributed to radiation, including one instance of brachial plexopathy in each dose group and one instance of radiation associated sarcoma in the dose escalated group.

## Discussion

Isolated loco-regional recurrence of breast cancer after mastectomy represents several clinical challenges. The current staging system has no mechanism to categorize these patients and treatment approaches are not standardized. Furthermore, these patients are more likely to develop additional loco-regional recurrences, as well as distant metastases, despite the best available therapy. Complete surgical resection has typically been associated with improved local control and overall survival [3,7,8,13], as has post-operative radiation therapy [7,9,14]. Systemic therapy is controversial, with at least one study showing no benefit to the addition of chemotherapy [15]. However, many studies appear to show improved outcomes with the addition of systemic chemotherapy [16,17] or hormonal therapy [10,18] in this setting. In the same vein, with improved chemotherapeutic options and patient selection, some patients will have a complete response (CR) to neoadjuvant systemic therapy. In these patients, data from this study suggest that the addition of surgical excision, with its concomitant morbidity, may not be necessary, adding another layer of clinical complexity in an already challenging disease.

Multi-modality therapy including systemic therapy, surgery and radiation has the potential to cure selected patients. In this study, which represents the largest single institutional study to date, we observed a 77% locoregional control rate and a 55% overall survival rate at 5 years. This again highlights that aggressive local-regional therapy is appropriate and compares favorably to the most recent experience in a similar group of patients [19]. We observed that the most important predictor for any outcome, including LRC, was the presence of residual gross disease at the time of radiation. Local failure was high at 38% in patients with clinically apparent residual disease and neither debulking surgery nor radiation dose escalation appeared to result in improved local control. Conversely, patients who achieved a complete response to chemotherapy and those with surgically resected disease had higher rates of loco-regional control.

We were dissatisfied with the overall loco-regional control rate in patients with isolated loco-regional recurrence from our last analysis of this patient cohort [2] and we speculated that dose escalation could be useful, as we had shown its value in inflammatory carcinoma [12]. However, in this study we could not demonstrate a benefit to a 10% dose escalation for isolated LRR in breast cancer. LRC rates in both the standard and dose escalated groups were similar with no specific subset of patients exhibiting a benefit.

Our strategy in treating patients with isolated loco-regionally recurrent breast cancer involves comprehensive – as opposed to involved field – post-operative radiotherapy in addition to systemic therapy. At least one study of LRR in breast cancer specifically comparing treatment of the tumor only compared to elective nodal radiation showed a significant improvement in local control with comprehensive irradiation [19]. In that study, multiple recurrences after radiation occurred outside the original area of recurrence. Here, however in our study all recurrences were in-field or at the field edge suggesting the appropriateness of the field selection and that intrinsic resistance is the primary cause for failure to control the disease.

In regards to the patient population as a whole, a number of indicators of poor prognosis were seen on univariate analysis, including nodal stage at the initial presentation as well as time to LRR after initial diagnosis. These factors are similar to those seen by ourselves and others [2,6], and thus provide further verification of their adverse effect on local control and survival outcomes. As in primary breast cancer, lower tumor and nodal stage, ER positivity, and smaller size of the recurrent tumor were all prognostic of better outcomes.

However, by far, the most significant prognostic factor in this study was residual, clinically detectable or gross disease at the time of radiation. Patients with gross disease at the time of radiation had dramatically poorer locoregional control and survival outcomes than those patients with either a complete surgical excision or a CR to chemotherapy and dose escalation had no significant effect on outcome. Interestingly, in patients with a CR to chemotherapy, locoregional control rates were similar to those in patients who were treated with a complete excision, implying surgical resection might be optional after complete response to chemotherapy. If further surgical resection is not an option, additional systemic therapies including targeted agents and radiation sensitizers represent the most promising approach for those that did not respond to chemotherapy and had gross residual disease at the time of radiation, especially since failure to achieve local control virtually guarantees that the patient will subsequently develop systemic metastasis.

This study is limited by several factors inherent in all retrospective reviews. Although the standard and dose escalated groups were well balanced in regards to most known factors, confounding factors may have influenced the total dose selection. In addition, only 159 total patients were reviewed. While this represents that largest study of its kind, this number may not allow the current study to detect small differences in the outcomes measured. Further, the difference in total dose between the two schedules is not profound. These results must be considered hypothesis generating; however given the unlikelihood of a randomized trial in this setting the high rates of local failure among patients with gross residual disease at the time of radiation certainly demonstrate room for improvement in treatment approach.

## Conclusions

Locoregionally recurrent breast cancer is a potentially curable disease. In this study of 159 patients we achieved 77% locoregional control and 55% overall survival at five years for the entire study group. In patients who could be rendered free from gross disease by surgery and/or chemotherapy the 5-year actuarial local control rate was 81% and the DFS rate was 50%. In the study population, radiation dose escalation to at least 66 Gy was not sufficient to achieve a clinically detectable improvement in loco-regional control rates. Most failures following radiation were in field and patients who did not achieve locoregional control of their disease had a dismal 16% DMFS at five years. Additionally, the presence of clinically or radiographically persistent disease at the time of radiation portends a very low probability of control despite aggressive local and systemic therapy. Since locoregional control remains an important objective, we recommend studies investigating the use of radiosensitizing agents current with radiation for patients who are not operative candidates or who have persistent disease following surgery or chemotherapy.

## Competing interests

The authors declare that they have no competing interests.

## Authors’ contributions

HS participated in the study design, recorded patient data, assisted in performing the statistical analysis and drafted the manuscript. ES participated in the study design, acquisition of patient data and in drafting the manuscript. SM participated in the study design, recorded patient data and assisted in drafting the manuscript. WW participated in the study design, acquisition of patient data and in drafting the manuscript. MG, GB, DB, FM and TB participated in the acquisition of patient data and the drafting of the manuscript. All authors read and approved the final manuscript.

## Supplementary Material

Additional file 1 **Table S1. **Univariate analysis of outcomes. Click here for file
