# Supplementary material for: Radiation dose escalation for loco-regional recurrence of breast cancer after mastectomy
Source: Radiat Oncol. 2013 Jan 11;8:13. doi: 10.1186/1748-717X-8-13 (PMC3552737; doi:10.1186/1748-717X-8-13)
Supplement: Additional file 1 — Table S1. Univariate analysis of outcomes. [file 1748-717X-8-13-S1.docx]

| **LRR after XRT Univariate** | **Comparison** | **RR** | **p-value** |  | **DFS after XRT Univariate** | **Comparison** | **RR** | **p-value** |
| --- | --- | --- | --- | --- | --- | --- | --- | --- |
|  |  |  |  |  |  |  |  |  |
| **Primary diagnosis** |  |  |  |  | **Primary diagnosis** |  |  |  |
| Percent Positive Nodes | Continuous | 1.01 | 0.02 |  | Percent Positive Nodes | Continuous | 1.02 | <0.001 |
| Nodal stage | N1-3 vs. N0 | 2.31 | 0.02 |  | Percent Positive Nodes | <25% vs. Negative | 2.16 | 0.003 |
| Tumor stage | T2-4 vs. T1 | 2.39 | 0.04 |  |  | ≥25% vs. <25% | 3.32 | <0.001 |
| Size | Continous | 1.14 | 0.01 |  | Nodal stage | N1-3 vs. N0 | 2.23 | <0.001 |
| LVSI | Positive vs. Negative | 3.00 | 0.01 |  | Tumor stage | T2-4 vs. T1 | 2.39 | <0.001 |
| ER status | Positive vs. Negative | 0.41 | 0.02 |  | Size | Continous | 1.14 | 0.01 |
| Grade | High vs. Low/Intermediate | 2.65 | 0.02 |  | LVSI | Positive vs. Negative | 1.70 | 0.03 |
|  |  |  |  |  | Grade | High vs. Low/Intermediate | 1.72 | 0.02 |
| **Recurrence** |  |  |  |  |  |  |  |  |
| Time to LRR | Continous | 0.99 | 0.02 |  | **Recurrence** |  |  |  |
| ER status | Positive vs. Negative | 0.55 | 0.03 |  | Time to LRR | Continous | 0.99 | 0.02 |
| Her 2 status | Positive vs. Negative | 2.16 | 0.05 |  | ER status | Positive vs. Negative | 0.55 | 0.03 |
| LRR size | ≥2 cm vs. < 2 cm | 1.61 | 0.03 |  | Her 2 status | Positive vs. Negative | 2.16 | 0.05 |
| LRR location | Other vs. Breast/CW only | 1.58 | 0.03 |  | LRR size | ≥2 cm vs. < 2 cm | 1.61 | 0.03 |
| Gross tumor at XRT | Yes vs. No | 2.28 | 0.03 |  | LRR location | Other vs. Breast/CW only | 1.58 | 0.03 |
| Resection | Yes vs. No | 0.48 | 0.05 |  | Nodal LRR | Yes vs. No | 1.60 | 0.03 |
|  |  |  |  |  | LRR resection | Yes vs. No | 0.54 | 0.01 |
| **OS after XRT Univariate** | **Comparison** | **RR** | **p-value** |  | Gross tumor at XRT | Yes vs. No | 2.35 | <0.001 |
|  |  |  |  |  |  |  |  |  |
| **Primary diagnosis** |  |  |  |  |  |  |  |  |
| Nodal stage | N1-3 vs. N0 | 2.55 | <0.001 |  | **DMFS after XRT Univariate** | **Comparison** | **RR** | **p-value** |
| Tumor stage | T2-4 vs. T1 | 2.92 | <0.001 |  |  |  |  |  |
| Size | Continuous | 0.99 | 0.02 |  | **Primary diagnosis** |  |  |  |
| LVSI | Positive vs. Negative | 2.23 | 0.01 |  | Nodal stage | N1-3 vs. N0 | 2.33 | <0.001 |
| Percent Positive Nodes | Continuous | 1.02 | <0.001 |  | Tumor stage | T2-4 vs. Tis-1 | 2.03 | 0.005 |
| Percent Positive Nodes | <25% vs. Negative | 2.62 | 0.001 |  | LVSI | Positive vs. Negative | 1.93 | 0.02 |
|  | ≥25% vs. <25% | 3.76 | <0.001 |  | Percent Positive Nodes | Continuous | 1.02 | <0.001 |
| ER status | Positive vs. Negative | 0.56 | 0.02 |  | Percent Positive Nodes | <25% vs. Negative | 2.50 | 0.001 |
|  |  |  |  |  |  | ≥25% vs. <25% | 3.36 | <0.001 |
| **Recurrence** |  |  |  |  |  |  |  |  |
| Time to LRR (mos) | Continous | 0.99 | 0.02 |  | **Recurrence** |  |  |  |
| Multiple LRR sites | Multiple vs. single | 1.94 | 0.005 |  | LRR location | Other vs. Breast/CW only | 1.63 | 0.04 |
| Size | ≥2 cm vs. <2 cm | 1.70 | 0.03 |  | LRR size | ≥2 cm vs. < 2 cm | 1.76 | 0.023 |
| ER status | Positive vs. Negative | 0.39 | 0.005 |  | Nodal LRR | Yes vs. No | 1.83 | 0.009 |
| Resection | Yes vs. No | 0.51 | 0.005 |  | Gross tumor at XRT | Yes vs. No | 2.41 | <0.001 |
| Gross tumor at XRT | Yes vs. No | 2.59 | <0.001 |  |  |  |  |  |
| Nodal LRR | Yes vs. No | 2.04 | 0.002 |  |  |  |  |  |
| LVSI | Positive vs. Negative | 2.75 | 0.05 |  |  |  |  |  |
| Subsequent LRR after  XRT | Yes vs. No | 2.39 | 0.001 |  |  |  |  |  |

**Supplemental table 1.** Univariate analysis of outcomes.
